# Contrasting Thermoelectric Transport Behaviors of *p*-Type PbS Caused by Doping Alkali Metals (Li and Na)

**DOI:** 10.34133/2020/4084532

**Published:** 2020-12-03

**Authors:** Zhenghao Hou, Dongyang Wang, Jinfeng Wang, Guangtao Wang, Zhiwei Huang, Li-Dong Zhao

**Affiliations:** ^1^School of Materials Science and Engineering, Beihang University, Beijing 100191, China; ^2^School of Physics, Henan Normal University, Xinxiang 453007, China

## Abstract

PbS is a latent substitute of PbTe thermoelectric materials, which is on account of its superiority in low cost and earth abundance. Here, the thermoelectric transport properties of *p*-type PbS by doping alkali metals (Na and Li) are investigated and it is verified that Li is a more effective dopant than Na. By introducing Li, the electrical and thermal transport properties were optimized collectively. The electrical transport properties were boosted remarkably via adjusting carrier concentration, and the maximum power factor (PF_max_) of ~11.5 *μ*W/cmK^2^ and average power factor (PF_ave_) ~9.9 *μ*W/cmK^2^ between 423 and 730 K in Pb_0.99_Li_0.01_S were achieved, which are much higher than those (~9.5 and ~7.7 *μ*W/cmK^2^) of Pb_0.99_Na_0.01_S. Doping Li and Na can weaken the lattice thermal conductivity effectively. Combining the enlarged PF with suppressed total thermal conductivity, a maximum ZT ~0.5 at 730 K and a large average ZT ~0.4 at 423-730 K were obtained in *p*-type Pb_0.99_Li_0.01_S, which are higher than ~0.4 and ~0.3 in *p*-type Pb_0.99_Na_0.01_S, respectively.

## 1. Introduction

The search for reliable and environmentally friendly new energy has attracted worldwide attention because of the shortage of fossil energy. A thermoelectric device is capable of transforming heat into electric energy immediately, which has shown great prospect in clean energy field [1–5]. The thermoelectric device efficiency is positively associated with the dimensionless figure of merit [6–9], ZT = *S*^2^*σT*/*κ*_tot_, where *T* represents absolute temperature, *S* expresses the Seebeck coefficient, *σ* denotes electrical conductivity, and *κ*_tot_ represents total thermal conductivity comprising electronic (*κ*_ele_) and lattice (*κ*_lat_) contributions [2, 10, 11].

Lead telluride- (PbTe-) based materials, as a kind of medium-temperature operating materials, have attracted extensive research interests on account of outstanding thermoelectric performance [12–14]. However, considering the high costs and low earth abundance of the Te element, the thermoelectric materials with rich resources should be developed. To date, one research hotpot in this field is to find an alternative material to substitute PbTe which possesses prominent thermoelectric properties [14–16]. As a similar alternative of PbTe, PbS possesses a NaCl structure and an alike band structure. Nevertheless, the poor electrical properties and large lattice thermal conductivity historically make PbS become an inferior thermoelectric material [11]. Aimed at solving the shortcomings of PbS, the approaches including carrier concentration optimization [17], band manipulation [18], and microstructure engineering [19–22] have been proved as effective strategies to manipulate electrical properties and thermal conductivity; the achievements realized through the above strategies well elucidate the potential performance of PbS.

Usually, doping is a powerful tactic to enhance ZT. Doping is essential in adjusting carrier concentration, and it is the prerequisite to gain a high ZT as all of those thermoelectric properties are interlinked by carrier concentration. On the assumption that the scattering or band structure is not modified obviously by a dopant, the Seebeck coefficient and electrical conductivity of degenerated semiconductor with a single parabolic band can be given using Equations (1) and (2) [23, 24].

(1) $$\begin{array}{c} \;S=\frac{8\pi^{2}k_{\text{B}}^{2}}{3eh^{2}}m^{*}T{\left(\frac{\pi }{3n}\right)}^{2/3}, \end{array}$$

(2) $$\begin{array}{c} \sigma =ne\mu_{n}, \end{array}$$

where *k*_B_ displays the Boltzmann constant, *e* shows the electron charge, *h* denotes the Planck constant, *m*^∗^ expresses effective mass, *n* is the carrier concentration, and *μ*_*n*_ represents the carrier mobility. Apparently, *S* and *σ* are in an inversely proportional relationship. Therefore, adjusting carrier concentration through balancing the relationship between *S* and *σ* is an important method to boost power factors. Doping with different elements may induce diverse impact on carrier concentration optimization and band structure manipulation. For example, *p*-type Na-doped PbS with CdS as second phases attains a large ZT ~1.3 at 923 K owing to extensive phonon scattering by nanophase precipitates and better electrical transport [25]; the ZT of *p*-type Tl-doped PbTe reaches ~1.5 at 773 K due to greatly enhanced Seebeck coefficients by deformation of electronic density of states [17]. It is meaningful to speculate the impacts by doping other alkali metals on thermoelectric performance in PbS.

In this article, we focused on PbS, which contains highly earth-abundant elements and owns higher melting point compared to PbTe. The thermoelectric properties in PbS doped by Li and Na were investigated systematically. The consequences reveal that the electrical and thermal properties were optimized synchronously through alkali metal doping. The electrical properties were improved through adjusting carrier concentration, and the PF_max_ of Pb_0.99_Li_0.01_S reached ~11.5 *μ*W/cmK^2^, which is far greater than ~9.5 *μ*W/cmK^2^ in Pb_0.99_Na_0.01_S. Both Li and Na can bring down the *κ*_lat_ because of point defects in PbS matrix. Li was more effective than Na in reducing *κ*_lat_ on account of larger mass and strain field fluctuations. Integrating enhanced PF and reduced *κ*_tot_, a higher ZT value ~0.5 at 730 K and average ZT ~0.4 at 423-730 K can be reached in Pb_0.99_Li_0.01_S, which are higher than ~0.4 and~0.3 in *p*-type Pb_0.99_Na_0.01_S, respectively, indicating that Li doping can improve thermoelectric performance of PbS more effectively than Na doping.

## 2. Experimental Section

### 2.1. Preparation Method

High-purity chemicals of Pb particle (99.99%), S (99.99%), Na (99.99%), and Li (99.99%) were weighed and loaded into carbon-coated quartz ampules under a N_2_-filled glove box. The ampules of the chemicals were evacuated under vacuum and flame-sealed. The pure chemicals were gradually warmed up to 723 K in 12 h, elevated to 1423 K in 7 h before keeping stable at 1423 K for 6 h, and finally naturally cooled to indoor temperature. The prepared specimens were pulverized and filtered with 400-mesh sieves for sintering through spark plasma sintering (*SPS-211LX*) using a pressure of 50 MPa at 923 K for 10 min.

### 2.2. Thermoelectric Properties

The acquired cylinder bulk materials were incised for measuring relevant thermoelectric properties. The *CTA* system was applied to measure electrical parameters (*S* and *σ*) at 300-730 K under He gas, and the samples were polished in a rectangular shape of 10 mm × 3 mm × 3 mm. The surfaces of measured samples were sprayed with thin-layer BN, which can inhibit volatilization and protect instrument [26]. The cylindrical disks with thickness of 1 mm and diameter of 6 mm were used to measure the thermal diffusivity (*D*). The thermal conductivity is computed through *κ*_tot_ = *DC*_p_*ρ*, and the thermal diffusivity was characterized using a *Netzsch LFA457* instrument with a laser flash method [27]. A thin graphite film on the surface of samples was utilized to cut down errors of emissivity for testing *D*. The density (*ρ*) was obtained based on mass and volume. All the densities of samples are around 7.2 g/cm^3^. The heat capacity (*C*_p_) was computed using the Debye model [28].

### 2.3. X-Ray Diffraction

The phase structure was investigated using an X-ray diffraction technique with *D/MAX2200pc* system with CuK*α* at 2*θ* = 20–80° (Rigaku, Japan, 40 kV, 40 mA). The scanning speed and step size of the XRD measurement are 6° min^−1^ and 0.02°, respectively.

### 2.4. Theoretical Calculations

The density functional theory (DFT) calculations were acquired through a projector-augmented wave (PAW) strategy [29, 30] with the Vienna Ab initio Simulation Package (VASP) [31]. The Perdew-Burke-Ernzerhof (PBE) exchange-correlation functional was used to model crystal and electronic structure. The used kinetic cutoff energy of plane waves is 500 eV. A 3 × 3 × 3 supercell (Pb_27_S_27_) was constructed to evaluate the defect formation energy of Li- (Pb_26_LiS_27_) and Na- (Pb_26_NaS_27_) doped systems. The internal coordinates of all atoms are entirely relaxed while the maximum residual ionic force is lower than 0.01 eV Å^−1^, and the total energy difference approaches 10^−6^ eV.

The formation energy of the defect *Q* (*Q* = Na, Li) in charge *q* is calculated by [32, 33]

(3) $$\begin{array}{c} ∆H^{\text{f}}\left(\text{PbS},Q^{q}\right)=E\left(\text{PbS},Q^{q}\right)-E\left(\text{PbS}\right)+\underset{i}{\sum }n_{i}\left(E_{i}+\mu_{i}\right)+q\left(E_{\text{F}}+E_{\text{V}}+∆V\right), \end{array}$$

where *E*(PbS, *Q*^*q*^) and *E*(PbS) refer to the total energy of defect *α* contained and undoped PbS in supercells with the same dimension, separately. *μ*_*i*_, *E*_*i*_, and *n*_*i*_ are the chemical potential, average energy of element in its most stable crystal structure, and the number of the atom *i* added to (*n*_*i*_ < 0) or taken from (*n*_*i*_ > 0) the host, respectively. *E*_F_ presents the Fermi level relative to energy location of valence band maximum (*E*_v_), which changes between 0 and band gap in PbS. The correction term Δ*V* is adopted to arrange the reference potential between the defect-containing and pure supercells with the same size [34]. All characteristic values are recombined to the 1s core level of the atom farthest from the defect [32, 35].

The formation energy depends on the chemical potential of each element, which is related to the off-stoichiometric degree (Pb- or S-rich condition). The different off-stoichiometric degrees will result in different chemical potential and formation energy. The upper and lower boundary chemical potential (*μ*_*i*_) is determined by the off-stoichiometric degree and the stability against precipitation of elemental Pb, S, Li, and Na:

(4) $$\begin{array}{c} \mu_{\text{Pb}},\mu_{\text{S}},\mu_{\text{Li}},\mu_{\text{Na}}\leq 0. \end{array}$$

The host compounds are obtained from the sum of the chemical potentials of Pb and S:

(5) $$\begin{array}{c} \mu_{\text{Pb}}+\mu_{\text{S}}=∆H^{\text{f}}\left(\text{PbS}\right), \end{array}$$

where Δ*H*^f^(PbS) is the formation energy of PbS in a rock-salt structure.

The second phase of Na_2_S, Li_2_S, and PbS_2_ should be avoided, and the corresponding formation energy should be larger than the sum of elemental chemical potential:

(6) $$\begin{array}{c} 2\mu_{\text{Na}}+\mu_{\text{S}}<∆H^{\text{f}}\left({\text{Na}}_{2}\text{S}\right),\\ 2\mu_{\text{Li}}+\mu_{\text{S}}<∆H^{\text{f}}\left({\text{Li}}_{2}\text{S}\right),\\ \mu_{\text{Pb}}+2\mu_{\text{S}}<∆H^{\text{f}}\left({\text{PbS}}_{2}\right). \end{array}$$

## 3. Results and Discussion

**Figure 1:**
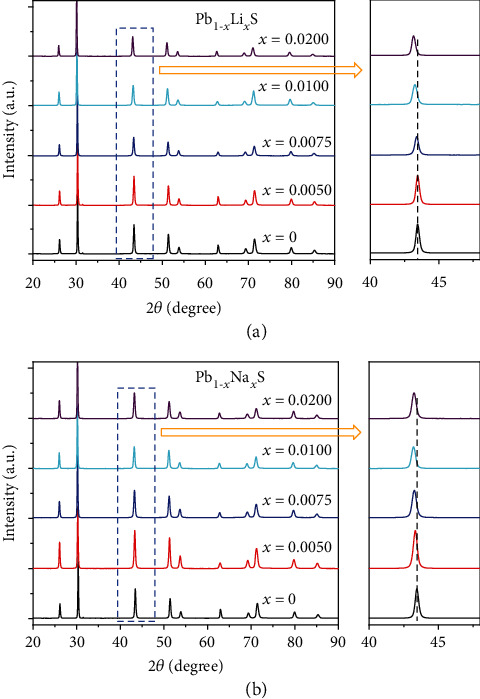
Powder XRD patterns of (a) Pb_1-*x*_Li*_x_*S and (b) Pb_1-*x*_Na*_x_*S.

Figure 1 demonstrates the detailed information of XRD results. All specimens possess a single phase of cubic PbS. The data peak transfers to low angle range as the Li and Na content was added, which indicates that Li and Na are doped into PbS lattice.

**Figure 2:**
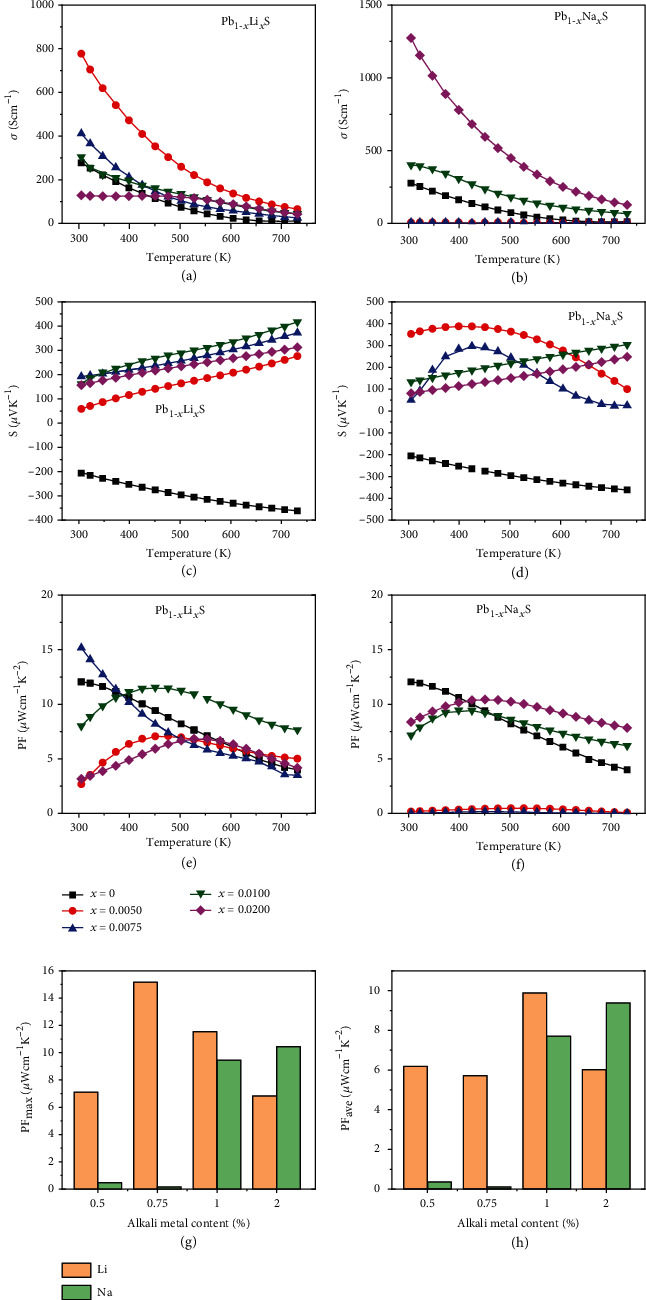
Temperature dependence of thermoelectric transport properties of Pb_1-*x*_Li*_x_*S and Pb_1-*x*_Na*_x_*S: (a, b) electrical conductivity, (c, d) Seebeck coefficient, (e, f) power factor (PF), (g) comparisons of maximum PF, and (h) averaged PF at 423-730 K.

Figure 2 depicts electrical properties in PbS with Li and Na doping. It can be clearly observed from Figures 2(a) and 2(b) that the *σ* falls off when temperature rises, except for low doping samples of Pb_1-*x*_Na*_x_*S (*x* = 0.005 and 0.0075).

For Li-doped samples, the *σ* possesses a tendency to increase first and then decrease with the stoichiometry of Li increasing and reaches to its maximum 777 S/cm in Pb_0.995_Li_0.005_S, as shown in Figure 2(a). However, the Na doping presents different results. As presented in Figure 2(b), the *σ* has an increased trend with increasing of Na content, and the maximum *σ* of 1274 S/cm can be realized in Pb_0.98_Na_0.02_S. The *σ* is positively correlated with *n*_H_ and *μ*_*n*_ from Equation (2), which are determined by the solid solubility and the different scattering mechanisms, respectively. The continued increase in *σ* of Pb_1-*x*_Na*_x_*S is mainly due to the fact that the higher solubility of Na than Li was caused by better ion radius matching (*r*_Li_^+^ = 0.76 Å, *r*_Na_^+^ = 1.02 Å, *r*_Pb_^2+^ = 1.26 Å).

As displayed in Figures 2(c) and 2(d), different from the undoped PbS, the Seebeck coefficients (*S*) for all doped samples are positive, indicating that Li and Na are effective *p*-type dopants in PbS. For Li-doped samples, the *S* present the trend of first decreasing and then increasing with an increasing Li content. For Na-doped samples, the trend is reversed that the *S* increase first and then decrease with an increasing Na content. These diametrically opposite trends reflect the contrary change of carrier concentration (*n*_H_) in those materials since the *S* are negatively correlated with *n*_H_.

**Figure 3:**
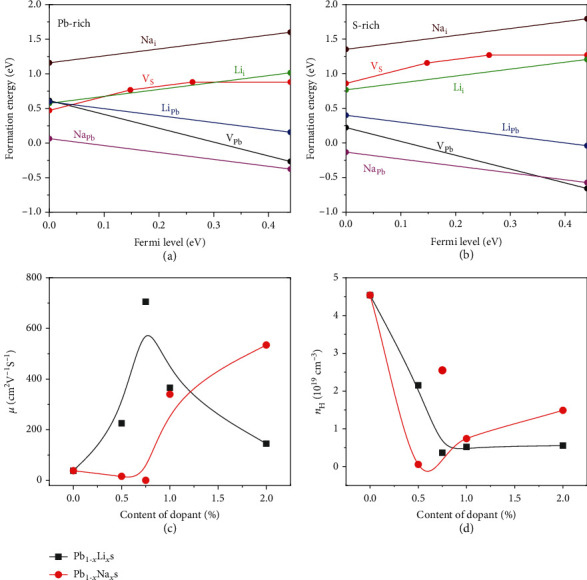
The calculated formation energy of potential defect in PbS under (a) Pb-rich and (b) S-rich conditions. The Fermi level with respect to the valence band maximum (VBM) of PbS. (c, d) The carrier mobility and carrier concentration of Pb_1-*x*_Li*_x_*S and Pb_1-*x*_Na*_x_*S at room temperature.

To evaluate the doping efficiency of Li and Na in PbS, the formation energy of potential defect was calculated and shown in Figures 3(a) and 3(b). The lower formation energy of Na_Pb_ indicates the spontaneous formation of Na_Pb_ in any conditions, which is even lower than that in V_Pb_. However, the Li_Pb_ has higher formation energy under Pb- and S- rich situations. In an equilibrium theory, the defect concentration can be evaluated by the formation energy Δ*H*, expressed as *n*_*i*_ = *N*_*i*_ × *e*^(−(*∆H*/*kT*))^ [36]. Thus, the larger formation energy of Li leads to a lower *n*_H_ and a larger *S* (Figure 2(c)).

As presented in Figure 2(e), for Li-doped samples, a higher PF can be obtained in Pb_0.99_Li_0.01_S in a broad temperature range, and the peak value can reach 11.5 *μ*W/cmK^2^ at 450 K. The peak PF for Pb_0.99_Li_0.01_S is much higher than Pb_0.99_Na_0.01_S which is ascribed to the lower *n*_H_, namely, adjusting carrier concentration to an optimized scope. Figures 3(c) and 3(d) show the carrier mobility and carrier concentration at room temperature which are calculated by the carrier effective mass of PbS (*m*^∗^ = 0.38 *m*_0_) [25]. According to Rowe and Bhandari's study [37], the *S* decreases and the *σ* increases as the *n*_H_ increases and the PF maximizes at a suitable *n*_H_ for a semiconductor. Therefore, adjusting the *n*_H_ to a reasonable range is the key factor to obtain higher PF. Compared with Pb_0.99_Na_0.01_S, Li doping leads to a relative lower *n*_H_ and higher PF. Figures 2(g) and 2(h) show the maximum power factor (PF_max_) at 300-730 K and average power factor (PF_ave_) within 423-730 K of *p*-type PbS samples. The PF_ave_ is calculated by Equation (7) in which the *T*_h_ and *T*_c_ are the temperatures of hot and cold ends. The PF_ave_ represents the overall capacity and level of electrical transports over a specified wide temperature range. The PF_max_ and PF_ave_ of the Pb_0.99_Li_0.01_S sample are 11.5 and 9.9 *μ*W/cmK^2^, respectively, higher than those of Pb_0.99_Na_0.01_S which are 9.5 and 7.7 *μ*W/cmK^2^. The present results reveal that the different dopants can reach the PF_max_ under their proper *n*_H_, which is strictly determined by the solid solubility of dopants in PbS.

(7) $$\begin{array}{c} {\text{PF}}_{\text{ave}}=\frac{1}{T_{\text{h}}-T_{\text{c}}}\int_{T_{\text{c}}}^{T_{\text{h}}}\text{PF}dT. \end{array}$$

**Figure 4:**
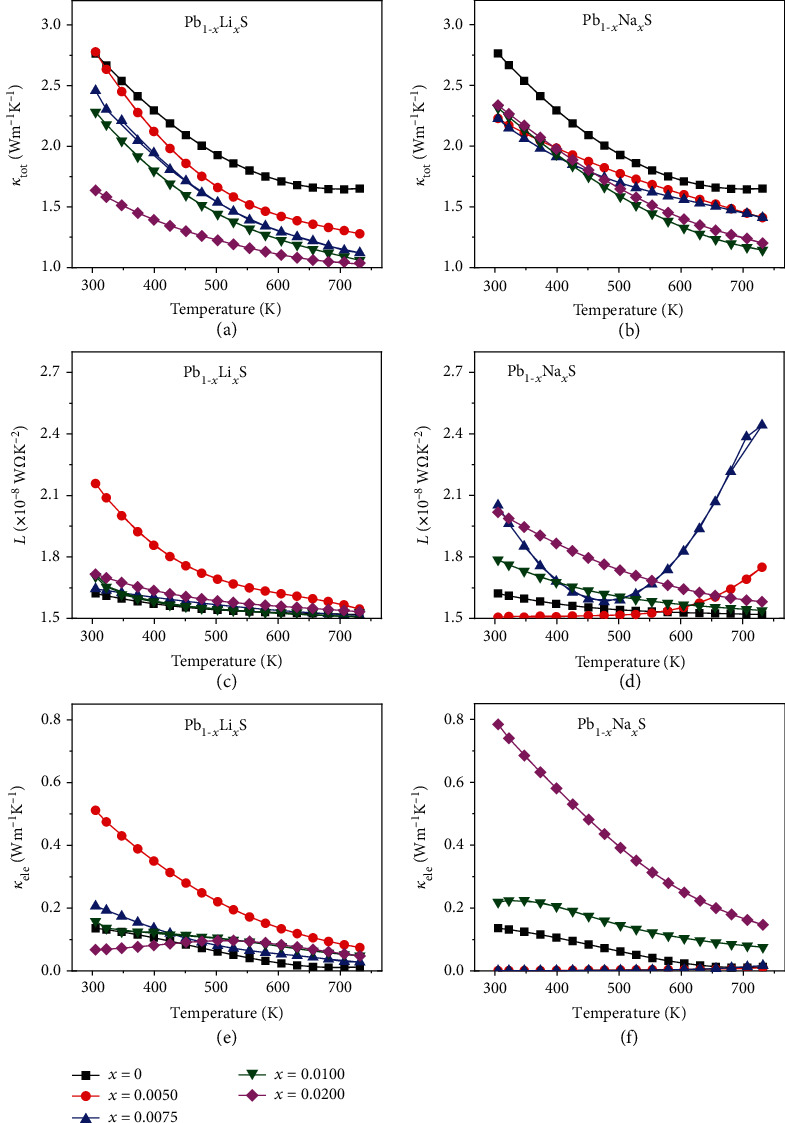
Temperature dependence of thermal transport properties of Pb_1-*x*_Li*_x_*S and Pb_1-*x*_Na*_x_*S: (a, b) total thermal conductivity, (c, d) Lorenz number, and (e, f) electronic thermal conductivity.

Figures 4(a) and 4(b) depict the *κ*_tot_ which decreases monotonically with the increase of temperature. The *κ*_tot_ of Pb_1-*x*_Li*_x_*S is lower than that in the undoped PbS, which is different from the larger content of Pb_1-*x*_Na*_x_*S since *σ* is higher. The *C*_p_ of Pb_0.99_Li_0.01_S and Pb_0.99_Na_0.01_S is 0.2094 J/g·K and 0.2092 J/g·K at 730 K, respectively. The *C*_p_ of Li-doped samples is similar to the *C*_p_ of Na-doped samples at the same content and temperature. The *κ*_tot_ includes lattice thermal conductivity and electronic thermal conductivity (*κ*_tot_ = *κ*_lat_ + *κ*_ele_) [22, 25], where the relationship between *κ*_ele_, *σ*, and Lorenz number (*L*) described in Equation (8) indicates that the *κ*_ele_ is proportional to *σ* [38, 39].

(8) $$\begin{array}{c} \kappa_{\text{ele}}=L\sigma T. \end{array}$$

The Lorenz number was obtained through calculating the Seebeck coefficient and integral chemical potentials [40]. Figures 4(c) and 4(d) show the Lorenz number in all samples as function of temperature. Higher *L* and *σ* lead to the larger *κ*_ele_ than those in undoped PbS, as revealed through Figures 4(e) and 4(f). Thus, the reduction in *κ*_tot_ is primarily caused by the decrease of *κ*_lat_.

**Figure 5:**
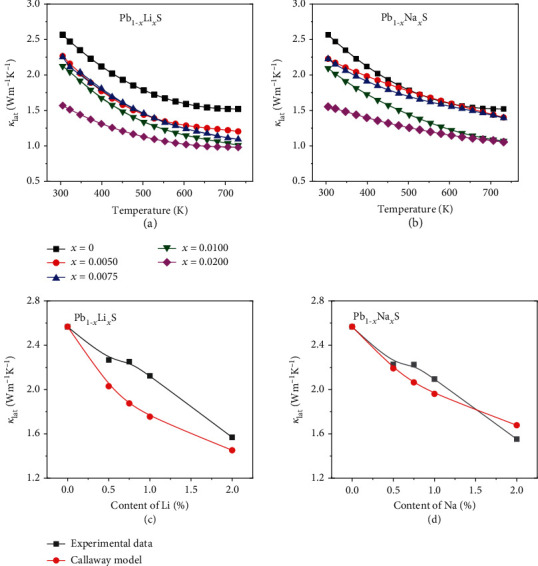
Temperature dependence of thermal transport properties of Pb_1-*x*_Li*_x_*S and Pb_1-*x*_Na*_x_*S: (a, b) lattice thermal conductivity and (c, d) comparisons of *κ*_lat_ between experimental and calculated values.

Figures 5(a) and 5(b) show that the *κ*_lat_ of all doped samples is lower than that of the undoped sample. The point defect scattering presumably reduces the *κ*_lat_ by Li and Na doping. Obviously, the *κ*_lat_ decreases with the increasing dopant content. More importantly, Li and Na are both effective in reducing the *κ*_lat_. To understand the phonon transports in Li- (Na) doped PbS, we adopted the Callaway model to evaluate point defect scattering caused by Li and Na doping [28, 41, 42].

When the temperature is higher than the Debye temperature, the point defect is an intensive scattering center to reduce the *κ*_lat_. According to the Callaway model [28, 42, 43], the ratio of the *κ*_lat_ between the defect-containing material and host material can be written as

(9) $$\begin{array}{c} \frac{\kappa_{\text{lat}}}{\kappa_{\text{lat},\text{p}}}=\frac{\tan^{-1}u}{u}, \end{array}$$

in which *κ*_lat_ and *κ*_lat,p_ represent the lattice thermal conductivities in doped and parent materials, separately. The parameter *u* is described using

(10) $$\begin{array}{c} u={\left(\frac{\pi^{2}\theta_{\text{D}}\Omega }{hv_{\text{a}}^{2}}K_{L,p}\Gamma \right)}^{1/2}, \end{array}$$

in which *h*, *Ω*, *v*_a_, and *θ*_D_ express the Planck constant, average atom volume, average sound velocity, and Debye temperature, separately. The imperfection scaling parameter (Γ) indicates that the phonon scattering intensity by atomic scale defects contains mass fluctuation Γ_M_ and strain field fluctuation Γ_S_. The phenomenological adjustable parameter (*ε*) regulates the uncertainty of Γ_S_. The imperfection scaling parameter Γ and the phenomenological adjustable parameter *ε* are expressed by the following equations [42]:

(11) $$\begin{array}{c} \Gamma =\Gamma_{\text{M}}+\varepsilon \Gamma_{\text{S}},\\ \varepsilon =\frac{2}{9}{\left(\frac{6.4\times \gamma \left(1+\nu_{\text{p}}\right)}{\left(1-\nu_{\text{p}}\right)}\right)}^{2}, \end{array}$$

where *ν*_p_ displays the Poisson ratio, which is calculated using the longitudinal (*ν*_l_) and transverse (*ν*_s_) acoustic velocities. The acoustic velocity of PbS was adopted in Poisson ratio and Grüneisen parameter (*γ*) calculation by the following equations:

(12) $$\begin{array}{c} \nu_{\text{p}}=\frac{1-2{\left(\nu_{\text{s}}/\nu_{\text{l}}\right)}^{2}}{2-2{\left(\nu_{\text{s}}/\nu_{\text{l}}\right)}^{2}},\\ \gamma =\frac{2}{3}\left(\frac{1+\nu_{\text{p}}}{2-3\nu_{\text{p}}}\right). \end{array}$$

When Pb sites are replaced by Li (Na), no change happens on the position of S, Γ_S_ = 0, which is defined by [44, 45]

(13) $$\begin{array}{c} \Gamma_{{\text{Pb}}_{1-x}Q_{x}\text{S}}=\frac{1}{2}{\left(\frac{M_{\left(\left(\text{Pb},Q\right)\right)}}{\bar{M}}\right)}^{2}\Gamma_{\left(\text{Pb},Q\right)},\\ \Gamma_{\left(\text{Pb},Q\right)}=\Gamma_{\text{M},\left(\text{Pb},Q\right)}+\varepsilon \Gamma_{\text{S},\left(\text{Pb},Q\right)},\\ \Gamma_{\text{M},\left(\text{Pb},Q\right)}=x\left(1-x\right){\left(\frac{∆M}{M_{\left(\text{Pb},Q\right)}}\right)}^{2}, \end{array}$$

where *∆M* = *M*_Pb_ − *M*_*Q*_, and *M*_(Pb, *Q*)_ = (1 − *x*)*M*_Pb_ + *xM*_*Q*_.

(14) $$\begin{array}{c} \Gamma_{\text{S},\left(\text{Pb},Q\right)}=x\left(1-x\right){\left(\frac{∆r}{r_{\left(\text{Pb},Q\right)}}\right)}^{2}, \end{array}$$

where *∆r* = *r*_*Pb*_ − *r*_*Q*_, and *r*_(Pb, *Q*)_ = (1 − *x*)*r*_Pb_ + *xr*_*Q*_.

Then,

(15) $$\begin{array}{c} \Gamma_{{\text{Pb}}_{1-x}Q_{x}S}=\frac{1}{2}{\left(\frac{M_{\left(\left(\text{Pb},Q\right)\right)}}{\bar{M}}\right)}^{2}x\left(1-x\right)\left[{\left(\frac{∆M}{M_{\left(\text{Pb},Q\right)}}\right)}^{2}+\varepsilon {\left(\frac{∆r}{r_{\left(\text{Pb},Q\right)}}\right)}^{2}\right]. \end{array}$$

The calculated mass fluctuations Γ_M,(Pb, *Q*)_ and strain field fluctuations Γ_S,(Pb, *Q*)_ have been given by Table 1. The higher deviations in atomic radius and mass between Pb and Li lead to larger *Γ*_(Pb,Li)_ than Γ_(Pb, Na)_, indicating more effective decreasing of *κ*_lat_ by Li doping. In Figures 5(c) and 5(d), the calculated results based on the Callaway model exhibit the same trend with the experimental data. The huge deviation may result from the formation of nanostructure even though in moderate doping concentration. This phenomenon confirms that Li and Na could both play effective roles in suppressing *κ*_lat_.

**Figure 6:**
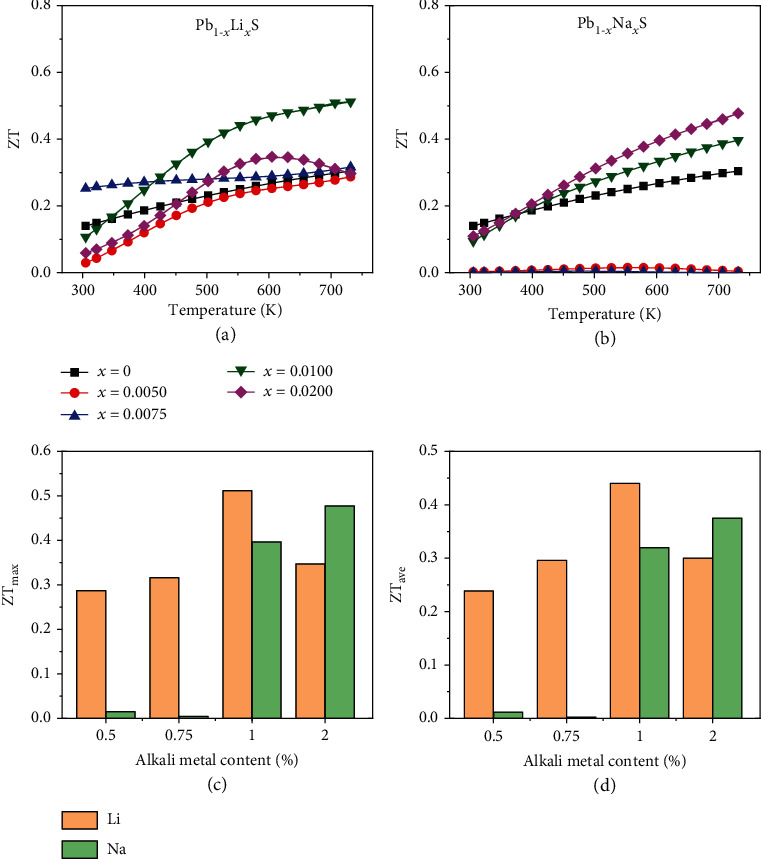
Temperature dependence of ZT of (a) Pb_1-*x*_Li*_x_*S and (b) Pb_1-*x*_Na*_x_*S and comparisons of (c) maximum ZT and (d) averaged ZT at 423-730 K.

The temperature-dependent ZT of PbS doped by Li and Na are presented in Figures 6(a) and 6(b). Pb_1-*x*_Li*_x_*S samples exhibit larger ZT than Pb_1-*x*_Na*_x_*S samples. The maximum ZT (ZT_max_) in Pb_0.99_Li_0.01_S attained ~0.5 when *T* = 730 K, which is higher than that in Pb_0.99_Na_0.01_S. The better thermoelectric performance of Li-doped samples is mainly due to the higher PF which results from the obtained proper *n*_H_ range and the slightly lower *κ*_tot_ from a more effective point defect scattering.

The variation trends of maximum ZT (ZT_max_) and average ZT (ZT_ave_), calculated by Equation (16), are consistent with PF_max_ and PF_ave_, as displayed in Figures 6(c) and 6(d). The ZT_max_ from room temperature to 730 K and ZT_ave_ within 423-730 K are 0.5 and 0.4 in Pb_0.99_Li_0.01_S, which is much higher than Pb_0.99_Na_0.01_S (0.4 and 0.3). The quality factor *B* is a parameter for estimating the optimal thermoelectric properties of a specific material according to the effective mass model, and the quality factor *B* is obtained by Equation (16). The weighted mobility *μ*_w_ is calculated by the electrical conductivity and Seebeck coefficient according to Equation (17) [46, 47].

(16) $$\begin{array}{c} B=9\frac{\mu_{\text{W}}}{\kappa_{\text{lat}}}{\left(\frac{T}{300}\right)}^{5/2}, \end{array}$$

(17) $$\begin{array}{c} \mu_{\text{W}}=\frac{3\sigma }{8\pi eF_{0}\left(\eta \right)}{\left(\frac{h^{2}}{2m_{e}k_{\text{B}}T}\right)}^{3/2}, \end{array}$$

in which *m*_*e*_ and *e* are unit mass of free electron and the electron charge, respectively. *F*_*n*_(*η*) represents the Fermi integral with *n* = 0 and is calculated by the following equations.

(18) $$\begin{array}{c} F_{n}\left(\eta \right)=\int_{0}^{\infty }\frac{x^{n}}{1+e^{x-\eta }}dx,\\ S=\pm \frac{k_{\text{B}}}{e}\left\{\frac{\left(r+5/2\right)F_{r+3/2}\left(\eta \right)}{\left(r+3/2\right)F_{r+1/2}\left(\eta \right)}-\eta \right\}, \end{array}$$

in which *r* shows the scattering factor and equals -1/2 here and *η* is the reduced chemical potential [46].

The calculated quality factors of Pb_0.99_Li_0.01_S and Pb_0.99_Na_0.01_S at 730 K are 0.4 and 0.2, respectively. The quality factor of Pb_0.99_Li_0.01_S is about twice higher than that of Pb_0.99_Na_0.01_S, so the ZT of Pb_0.99_Li_0.01_S is higher, which is caused by the enhanced PF by adjusting *n*_H_ in a reasonable range. The thermoelectric conversion efficiencies are calculated by Equation (20) [28]:

(19) $$\begin{array}{c} {\text{ZT}}_{\text{ave}}=\frac{1}{T_{\text{h}}-T_{\text{c}}}\int_{T_{\text{C}}}^{T_{\text{h}}}\text{ZT}dT, \end{array}$$

(20) $$\begin{array}{c} \eta =\frac{T_{\text{h}}-T_{\text{c}}}{T_{\text{h}}}\frac{\sqrt{1+{\text{ZT}}_{\text{ave}}}-1}{\sqrt{1+{\text{ZT}}_{\text{ave}}}+\left(T_{\text{c}}/T_{\text{h}}\right)}, \end{array}$$

in which *T*_h_ and *T*_c_ represent the temperature in hot and cold end, respectively. The maximum calculated thermoelectric conversion efficiency based on single leg is ~4.8% in Pb_0.99_Li_0.01_S which is higher than Pb_0.99_Na_0.01_S (~3.4%) when *T*_h_ = 730 K and *T*_c_ = 423 K, indicating Li is a valid dopant to regulate the thermoelectric performance through tuning *n*_H_.

## 4. Conclusion

This work indicates that Li doping is more effective than Na doping in thermoelectric performance optimization in PbS. The boosted thermoelectric performance of Li-doped PbS is completed by enhancing the PF through regulating *n*_H_ in a reasonable range. The PF_max_ and PF_ave_ between 423 and 730 K of Pb_0.99_Li_0.01_S reached ~11.5 and~9.9 *μ*W/cmK^2^, which are much better compared with ~9.5 and ~7.7 *μ*W/cmK^2^ of Pb_0.99_Na_0.01_S. Pb_1-*x*_Li*_x_*S samples possess slightly smaller *κ*_lat_ than that of Pb_1-*x*_Na*_x_*S because of larger mass and strain field fluctuations. At last, higher *Z*T_max_ ~0.5 at 730 K and ZT_ave_ ~0.4 at 423 K-730 K can be obtained in Pb_0.99_Li_0.01_S. The calculated thermoelectric conversion efficiency ~4.8% is achieved in Pb_0.99_Li_0.01_S with *T*_h_ = 730 K and *T*_c_ = 423 K. In the future, the ZT for Li-doped PbS can also be raised through nanostructuring, manipulating band structures, and other approaches.

## Figures and Tables

**Table 1:** Calculated imperfection scaling parameters and *κ*_lat_ (W/mK) of Li- and Na-doped PbS based on the Callaway model.

Samples	Γ_M,(Pb, *Q*)_	Γ_S,(Pb, *Q*)_	Γ_(Pb, *Q*)_	*κ*_lat_
Pb_0.995_Li_0.0050_S	0.004693	0.000072	0.010254	2.03
Pb_0.995_Li_0.0075_S	0.007055	0.000107	0.015382	1.87
Pb_0.99_Li_0.0100_S	0.009429	0.000143	0.020510	1.75
Pb_0.98_Li_0.0200_S	0.019038	0.000284	0.041028	1.45
Pb_0.995_Na_0.0050_S	0.003967	0.000042	0.007189	2.19
Pb_0.995_Na_0.0075_S	0.005962	0.000062	0.010780	2.06
Pb_0.99_Na_0.0100_S	0.007965	0.000083	0.014370	1.96
Pb_0.98_Na_0.0200_S	0.016056	0.000163	0.028714	1.68
